# The Prevalence and Distribution of Osteopathic Chief Residents in Emergency Medicine

**DOI:** 10.7759/cureus.54697

**Published:** 2024-02-22

**Authors:** Emma Wandro, Sahar Rammaha, Kevin McGurk

**Affiliations:** 1 Department of Emergency Medicine, Medical College of Wisconsin, Milwaukee, USA; 2 Emergency Medicine, Harbor-University of California Los Angeles Medical Center, Los Angeles, USA

**Keywords:** chief resident, emergency medicine residency, osteopathic medical graduate, emergency medicine training, chief resident selection, osteopathic

## Abstract

Background

The chief resident position fulfills important administrative and educational functions for emergency medicine (EM) residency programs and has been associated with advanced academic and career opportunities. This study seeks to determine the prevalence and distribution of Doctor of Osteopathic Medicine (D.O.) and allopathic Doctor of Medicine (M.D.) degrees among chief residents within EM residencies.

Methodology

EM residency program websites, affiliated social media accounts on X (formerly Twitter) and Instagram, and program coordinator surveys were used to collect data, including the number of current residents, chief residents, and the listed medical degrees for residents and program directors during the 2021-2022 and 2022-2023 academic years. A Pearson’s chi-square test was used to compare the number of residents, chief residents, and program directors by medical degree.

Results

A total of 188/229 (82.1%) and 201/229 (87.8%) eligible EM residencies identified their current chief residents and the medical degrees of their residents for the academic year 2021-2022 and 2022-2023, respectively. Of 14,487 EM residents included during the study period, 3,676 (25.4%) were D.O.s, and of the 1,230 chief residents identified, 362 (29.4%) were D.O.s.

Conclusions

The proportion of D.O. chief residents was higher than the proportion of D.O. residents within EM residencies. However, osteopathic residents were asymmetrically distributed across programs and were most likely to serve as chief residents at programs with a higher proportion of D.O. trainees and at programs with osteopathic program directors.

## Introduction

The chief resident position fulfills important administrative and educational functions for emergency medicine (EM) residency programs [1-3]. Residents serving in this role often receive additional training and hone leadership and administrative skills that are beneficial to their career pursuits [2]. The position helps prepare trainees for future academic roles and is associated with an increased likelihood of a career in academic medicine [2,4]. Chief residents are typically selected based on multiple factors, including program director, faculty, and co-resident input. While prior studies have examined demographics, including gender and age among chief residents, little is known about the distribution between those with Doctor of Osteopathic Medicine (D.O.) and allopathic Doctor of Medicine (M.D.) degrees among this cohort [4-7]. Given the impact of the chief resident role on future academic and career prospects, a disparity between allopathic and osteopathic chiefs could contribute to broader representation issues within specialty leadership and academia. This study seeks to determine the prevalence and distribution of osteopathic chief residents within EM residencies.

## Materials and methods

We performed a retrospective observational study evaluating the number of osteopathic and allopathic residents, chief residents, and program directors in EM residencies across the United States. The Society for Academic Emergency Medicine program directory was used to compile a list of EM residencies accredited by the Accreditation Council for Graduate Medical Education for the 2021-2022 and 2022-2023 academic years. Programs that are no longer operational, new programs without a senior class of residents, and international programs were excluded from the study. Publicly available program websites and their affiliated social media accounts onX (formerly Twitter) and Instagram were used to collect data, including the number of current residents, chief residents, and the listed medical degrees for residents, chief residents, and program directors during the 2021-2022 and 2022-2023 academic years. When unlisted, program coordinators were surveyed for the same information.

After a brief training to standardize collection, data acquisition occurred between November 30, 2021, and December 31, 2021, and again over the same period for 2022. Dates were selected to follow the typical periods for chief selection (traditionally in the spring) and the summer transition of incoming and graduating residents. Reliability between the two observers was assessed on a 20-program sample using Cohen’s kappa. Linear regression analysis was used to assess the association between the proportion of D.O. residents within a program and the number of D.O. chief residents within a program. Subgroup analysis was then performed by separating programs by their proportion of D.O. residents into discrete quintiles (0-20%, 21-40%, 41-60%, 61-80%, and 81%-100%) and comparing the proportion of D.O. chiefs to D.O. residents by quintile.

Descriptive statistics and Pearson’s chi‐square test were performed using Excel (Microsoft Corporation, Redmond, WA, USA). Statistical significance was defined as p-values ≤0.05. This study was exempted from review by the Medical College of Wisconsin Institutional Review Board.

## Results

Interobserver agreement was excellent with a kappa coefficient of 1.0. A total of 188/229 (82.1%) and 201/229 (87.8%) eligible EM residencies identified their current chief residents and the medical degrees of their trainees for the 2021-2022 and 2022-2023 academic years, respectively. Of the 14,487 EM residents included, 3,676 (25.4%) were osteopathic physicians. The median number of chief residents per program was three with a range from one to seven. Of the 1,230 chief residents identified, 362 (29.4%) were D.O.s.

A total of 37/188 (19.6%) and 40/201 (19.9%) of the included program directors for the 2021-2022 and 2022-2023 academic years, respectively, were osteopathic physicians. Among programs with M.D. program directors, 215/1,035 (20.8%) of chief residents and 2,188/12,371 (17.7%) of total residents were D.O.s. At programs with D.O. program directors, 147/195 (75.4%) of chief residents and 1,488/2,116 (70.3%) of total residents were osteopathic physicians.

The proportion of D.O. chief residents was higher than both the proportion of D.O. residents (29.4% vs. 25.4%, p = 0.002) and D.O. program directors (29.4% vs. 19.8%, p <0.001) in EM residencies.

The proportion of D.O. residents, chief residents, and program directors was similar between the 2021-2022 and 2022-2023 cohorts, as shown in Table 1.

**Table 1:** Trend in osteopathic residency leadership in emergency medicine 2021-2023. ^a^: P-values from the chi-square test of independence. Statistical significance is defined by p-values ≤0.05.

	2021–2022	2022–2023	P-value^a^
Included programs	188/229 (82.1%)	201/229 (87.8%)	0.0895
Osteopathic residents	1,776/7,051 (25.2%)	1,900/7,436 (25.6%)	0.6153
Osteopathic chief residents	182/593 (30.7%)	180/637 (28.3%)	0.3493
Osteopathic program directors	37/188 (19.7%)	40/201 (19.9%)	0.9567
Programs without an osteopathic resident	33/188 (17.6%)	30/201 (14.9%)	0.4820

**Figure 1:**
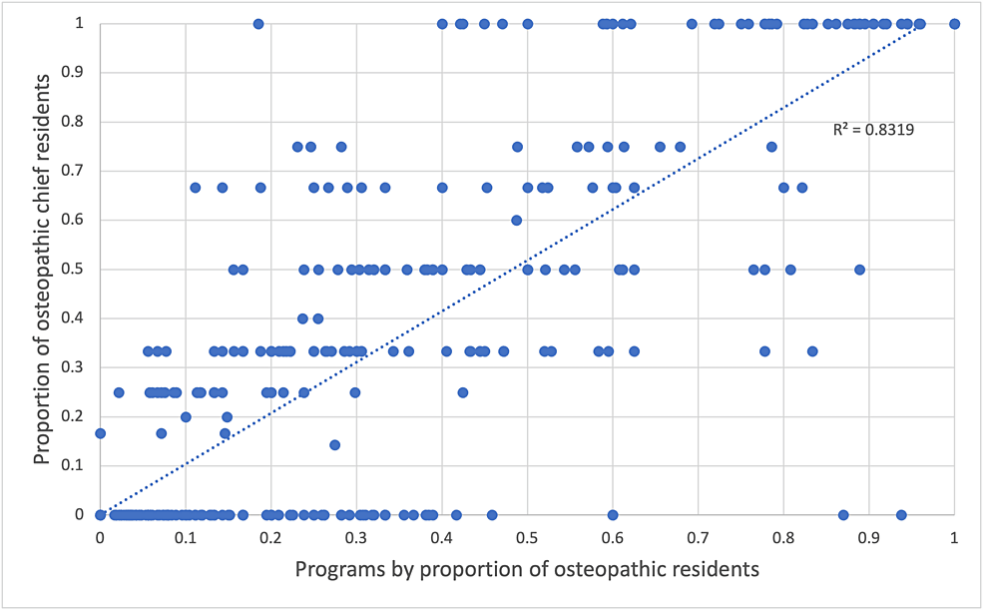
Distribution of osteopathic chief residents 2021-2023.

The proportion of osteopathic chief residents within a program largely mirrored the proportion of D.O. residents in those programs, as shown in Figure 1. Linear regression yielded a coefficient of determination, r^2^ = 0.83 (r = 0.91; p < 0.0001).

When stratified into quintiles by the proportion of the residency comprising D.O.s, the percentage of osteopathic chief residents was marginally higher than the percentage of osteopathic residents in all but the highest quintile. More than half of all programs had resident rosters with fewer than 20% osteopathic residents. Among these programs, 483/8,325 (5.8%) of residents and 53/670 (7.9%) of chief residents were D.O.s. In both academic years studied, at least 30 programs included no osteopathic trainees. At residencies comprising 21-40% osteopathic residents, 726/2,517 (28.8%) of residents and 69/232 (29.7%) of chief residents were D.O.s. At programs with 41-60% osteopathic trainees, 819/1,632 (50.2%) of residents and 81/143 (56.6%) of chief residents were D.O.s. At those with 61-80% osteopathic trainees, 628/901 (69.7%) of residents and 63/80 (78.6%) of chief residents held osteopathic degrees. At programs with more than 80% D.O.s, 1,006/1,094 (92.0%) of residents and 94/103 (91.3%) of chief residents held osteopathic degrees.

Most programs excluded for missing data only failed to identify their chief residents. For 2022-2023, 21 of 28 programs with incomplete data listed the degrees of their trainees and program directors but not their chief residents. Collectively, D.O.s comprised a similar percentage of program directors (19.0% vs. 19.9%) and a modestly increased proportion of residents (33.2% vs. 25.6%) at the excluded versus included programs, respectively.

## Discussion

The proportion of D.O.s serving in the role of chief resident is higher than the overall proportion of EM residents with osteopathic degrees. At residencies with D.O. program directors, trainees and chief residents are more likely to be osteopathic physicians. Unsurprisingly, residencies with a larger proportion of osteopathic residents have a larger proportion of osteopathic physicians as chief residents.

The results demonstrate that osteopathic residents are no less likely to be selected as chief residents than their allopathic peers. However, the distribution of osteopathic residents across programs is decidedly asymmetric and supports the notion that certain programs are *D.O. friendly* while others are not [8-11]. More than half of all EM trainees are educated in programs with <10% D.O.s, including at least 30 residencies with no osteopathic residents. D.O.s training in this cohort of programs were overrepresented in the chief role (7.9% of chief residents vs. 5.8% of residents overall). This suggests that for many D.O.s, a limiting factor may not be finding success within a program but rather matching into it. While the percentage of programs with no D.O. trainees decreased from 2021-2022 to 2022-2023, the change was nominal (17.6% to 14.9%, p = 0.482). As the national landscape changes and the overall popularity and competitiveness of the specialty evolves, so too may the prevalence of osteopathic physicians in residency positions and leadership roles. The striking decrease in EM applicants seen for the 2023 match cycle may compel programs with few or no D.O. residents to re-evaluate how they evaluate and rank these applicants [12].

While there are slight differences between osteopathic and allopathic EM resident U.S. Medical Licensing Examination (USMLE) Step 1 scores (mean of 228 and 233, respectively), this is unlikely to fully explain the degree of variable osteopathic representation across residencies [13,14]. Several other factors likely contribute, including applicant self-selection, access to appropriate mentorship, and availability of *home* EM rotations affiliated with applicants’ medical schools [9-11]. Prior studies have also indicated some programs do not match osteopathic applicants due to concerns about the quality of their medical education or how osteopathic trainees might impact the reputation of the program [9].

Although the chief resident role has been associated with future academic medical careers, it is unclear whether this is as true for osteopathic trainees. While serving as chief resident may afford an individual more academic opportunities, a wide gap persists in traditional post-residency metrics for success in academic medicine between D.O.s and M.D.s. Though osteopathic physicians comprised 29.4% of chief residents in our study, they make up <1% of editorial positions for major medical journals, <3% of authors in major EM journals, and <3% of major EM research grant recipients [15-18]. The factors contributing to this gap, despite the proportional representation of D.O.s as chief residents, are unknown.

Limitations

This study relied upon publicly available data and/or program coordinator survey responses. As some residencies did not report all necessary information, a mean of 14.8% of programs were excluded from the analysis. While we do not believe this meaningfully changes the conclusions drawn, a fuller representation of EM programs may have altered some statistical considerations.

Our study compared M.D. and D.O. representation but could not make a distinction between graduates of U.S. medical schools and international medical graduates (IMGs) with M.D.s. IMGs face larger challenges matching and have lower mean USMLE scores than U.S. medical graduates. Residents with non-D.O. or -M.D. degrees (i.e., M.B.B.S.) were excluded from the analysis but make up an exceedingly small portion of current EM trainees nationwide [19]. We believe this is unlikely to have skewed results.

## Conclusions

Osteopathic residents are asymmetrically distributed across EM residencies with program composition ranging from no D.O.s to 100% D.O. residents. Unsurprisingly, D.O.s are most likely to serve as chief residents at programs with a higher proportion of D.O. trainees and at programs with osteopathic program directors. However, the proportion of D.O. chief residents was higher than the proportion of D.O. residents within EM training programs. This was true even at programs with few D.O. residents, suggesting that for many D.O.s a limiting factor may not be finding success within a program but rather matching into it. Despite proportional over-representation in the chief resident role, D.O.s remain under-represented in other academic leadership roles and as recipients of grant funding. This disparity warrants further investigation.

## References

[1] VanOrder T, Wisniewski SJ (2018). Chief resident skills: a study on resident perceptions of skill importance and confidence. Spartan Med Res J.

[2] Hafner JW, Gardner JC, Boston WS, Aldag JC (2010). The chief resident role in emergency medicine residency programs. West J Emerg Med.

[3] Turner J, Litzau M, Mugele J, Pettit K, Sarmiento EJ, Humbert A (2020). Qualities important in the selection of chief residents. Cureus.

[4] Jordan J, Hopson LR, Clarke SO (2021). Academic springboard: the chief resident position correlates with career path in emergency medicine. AEM Educ Train.

[5] Farrell KJ, Walker LE, Battaglioli N, Heaton HA, Lohse C, Sadosty AT (2021). Six years of gender equity in emergency medicine chief resident selection. AEM Educ Train.

[6] Saak JC, Mannix A, Stilley J, Sampson C (2021). Diversity begets diversity: factors contributing to emergency medicine residency gender diversity. AEM Educ Train.

[7] Mannix A, Parsons M, Krzyzaniak SM, Black LP, Alvarez A, Mody S, Gottlieb M (2020). Emergency Medicine Gender in Resident Leadership Study (EM GIRLS): the gender distribution among chief residents. AEM Educ Train.

[8] Beckman JJ, Speicher MR (2020). Characteristics of ACGME residency programs that select osteopathic medical graduates. J Grad Med Educ.

[9] Pelletier-Bui AE, Schrepel C, Smith L (2020). Advising special population emergency medicine residency applicants: a survey of emergency medicine advisors and residency program leadership. BMC Med Educ.

[10] Stobart-Gallagher M, Smith L, Giordano J, Jarou Z, Lutfy-Clayton L, Kellogg A, Hillman E (2019). Recommendations from the Council of Emergency Medicine Residency Directors: osteopathic applicants. West J Emerg Med.

[11] Stobart-Gallagher M, O'Connell A (2018). Preparing osteopathic students for the single graduate medical education accreditation system: evaluating factors for match success in emergency medicine. West J Emerg Med.

[12] (2023). Interest in emergency medicine residencies drops. https://www.bloomberg.com/news/newsletters/2023-04-04/interest-in-emergency-medicine-residencies-drops.

[13] (2021). Charting outcomes in the match: senior students of U.S. DO medical schools. https://www.nrmp.org/wp-content/uploads/2020/07/Charting-Outcomes-in-the-Match-2020_DO-Senior_final.pdf.

[14] (2021). Charting outcomes in the match: senior students of U.S. MD medical schools. https://www.nrmp.org/wp-content/uploads/2020/07/Charting-Outcomes-in-the-Match-2020_MD-Senior_final.pdf.

[15] Ashurst JV, Galuska M (2016). Osteopathic physicians on the editorial boards of major medical journals over the past 30 years. J Am Osteopath Assoc.

[16] Lammers R, Simunich T, Ashurst J (2016). Authorship trends of emergency medicine publications over the last two decades. West J Emerg Med.

[17] Gordon RD, Kwon NS, Levy PD, Madsen TE, Greenberg MR (2020). Evaluating the diversity of Emergency Medicine Foundation (EMF) grant recipients in the last decade. West J Emerg Med.

[18] Antony M, Savino J, Ashurst J (2017). Difference in R01 grant funding among osteopathic and allopathic emergency physicians over the last decade. West J Emerg Med.

[19] (2022). Results and data: 2020 main residency match. https://www.nrmp.org/wp-content/uploads/2021/12/MM_Results_and-Data_2020-rev.pdf.

